# Breed-Associated Roasted Aroma Signatures Across Eight Chinese Sheep Breeds: Integrating Fatty Acid Composition, Volatile Profiles, and Aroma-Active Compounds

**DOI:** 10.3390/foods15213873

**Published:** 2026-10-29

**Authors:** Bin Chen, Jinlong Li, Wujun Liu

**Affiliations:** College of Animal Science, Xinjiang Agricultural University, Urumqi 830052, China; chenbin_1998@163.com (B.C.); lijinlong0120@126.com (J.L.)

**Keywords:** lamb, roasting, fatty acids, volatile compounds, GC–O

## Abstract

Breed-associated differences in roasted-lamb flavour are often described from volatile profiles alone, whereas the links among lipid precursors, volatile abundance and olfactory activity remain less clear. Here, fatty acid profiling, headspace solid-phase microextraction–gas chromatography–mass spectrometry (HS-SPME–GC–MS) and gas chromatography–olfactometry–mass spectrometry (GC–O–MS) were combined to compare eight Chinese sheep breeds under standardised roasting conditions, although rearing conditions differed among groups. Major unsaturated fatty acids, particularly C18:1n-9, C18:2n-6, C18:3n-3 and C20:4n-6, differed markedly among breeds. Aldehydes and pyrazines dominated the roasted volatilome, but their relative contributions varied. GC–O–MS revealed a shared roasted/nutty aroma backbone, with 2,3-dimethylpyrazine detected across all breeds, whereas lipid-derived odorants including hexanal, nonanal, octanal, (*E*)-2-octenal and 1-octen-3-ol showed greater breed-associated variation. Some breeds exhibited pyrazine-dominant profiles, whereas others showed greater contributions from lipid-derived aldehydes and alcohols. Unsaturated fatty acids were more closely associated with lipid-derived volatiles than with pyrazines. These findings indicate that breed-associated differences in roasted-lamb aroma are characterised mainly by shifts in the relative contribution of lipid oxidation products and Maillard-derived odorants against a shared aroma-active background.

## 1. Introduction

Lamb flavour is an important determinant of eating quality and consumer acceptance, and substantial variation has been reported among sheep breeds and production systems. China possesses diverse indigenous sheep genetic resources, many of which are valued for distinctive meat-quality characteristics. Comparative studies have shown that sheep breeds differ in their volatile profiles, particularly in aldehydes, alcohols, ketones and other aroma-related compounds [1,2]. Similar variation has been reported among Tibetan sheep breeds, where hexanal, octanal, nonanal, 1-octen-3-ol and 2-pentylfuran contributed strongly to differences among breeds and muscle types [3]. These findings suggest that breed-associated differences in lamb flavour are reflected, at least in part, in the composition of the volatile fraction.

Roasting promotes both lipid oxidation and Maillard reactions, which together shape the characteristic aroma of lamb. Unsaturated fatty acids can generate aldehydes, alcohols, ketones and furans during thermal oxidation, whereas amino acids and reducing sugars contribute pyrazines, sulfur-containing compounds and other heterocyclic volatiles through Maillard chemistry [4]. Changes in muscle lipid composition can therefore influence the formation of lipid-derived volatile compounds during heating [5], and associations between individual unsaturated fatty acids and characteristic lamb volatiles have been reported [6]. Fatty acid composition consequently provides an important precursor background for roasted aroma formation, although precursor abundance alone does not determine the final volatile profile.

Most comparisons among sheep breeds have relied primarily on volatile abundance measured by gas chromatography–mass spectrometry (GC–MS) or gas chromatography–ion mobility spectrometry (GC–IMS). However, chemical abundance does not necessarily reflect olfactory importance because volatile compounds differ greatly in odour threshold and quality. Gas chromatography–olfactometry (GC–O) provides complementary information by identifying compounds perceived at the olfactory detection port. GC–MS/O and odour-activity analysis have identified a restricted group of aldehydes, ketones, furans and other compounds contributing to characteristic mutton aroma [7], while recent aroma-recombination studies have shown that only a subset of the volatile pool is required to reproduce major roasted-lamb aroma attributes [8]. Carbonyl compounds, particularly aldehydes such as hexanal, also increase markedly during lamb roasting [9]. These observations underline the importance of considering both volatile abundance and olfactory activity when characterising roasted-lamb aroma.

Whether breed-associated variation in roasted-lamb aroma is accompanied by corresponding differences in lipid precursors and aroma-active compounds remains less well defined. We hypothesised that variation in muscle fatty acid composition would be associated with differences in lipid-derived volatile profiles, while Maillard-derived odorants would provide a common roasted aroma background across the sampled breeds. In the present study, five Xinjiang and three non-Xinjiang sheep breeds were compared for fatty acid composition, roasted volatile profiles and GC–O-active compounds. GC–MS and GC–O were combined to characterise the chemical and olfactory features of the roasted volatilome, and fatty acid–volatile associations were examined to determine how variation in lipid composition was related to reaction-associated volatile patterns. Particular attention was given to the shared aroma-active background across breeds and to differences in the relative contributions of lipid-derived and Maillard-derived compounds. As the animals originated from different regions and routine production systems, the study examined breed-associated patterns rather than independent genetic breed effects.

## 2. Materials and Methods

### 2.1. Animals and Sample Collection

All procedures involving sample collection were approved by the Experimental Animal Welfare and Ethics Committee of Xinjiang Agricultural University (approval no. 2023053). Animal-related procedures were conducted in accordance with ISO/TS 34700:2016 [10]. This study did not involve an experimental feeding or intervention trial.

A total of 48 healthy male lambs representing eight sheep breeds were included, with six animals per breed: Lop Nur (LB), Kazakh (HSK), Duolang (DL), Altay (ALT), Turpan Black (TLF), Mongolian (MG), Tan (TY) and Hu (HY) sheep. All animals were approximately 12 months of age and were sampled at a comparable commercial production stage. Each breed was represented by six lambs from a single local purebred farm, with no known close familial relationships among the selected animals. No experimental diet, feed additive or other management intervention was imposed by the authors before slaughter; the animals remained under the routine production systems of their respective source farms. To minimise variation unrelated to breed-origin group, only clinically healthy males of the same age were included, and the same muscle, post mortem sampling, storage and subsequent analytical procedures were used for all animals. Because breed and pre-slaughter production conditions were not experimentally separated, observed differences cannot be attributed solely to genetic breed effects. Although six animals were included per breed, the study identified distinct patterns in fatty acid composition and roasted volatile profiles. Further studies involving larger populations would help establish the consistency of these findings.

**Figure 1:**
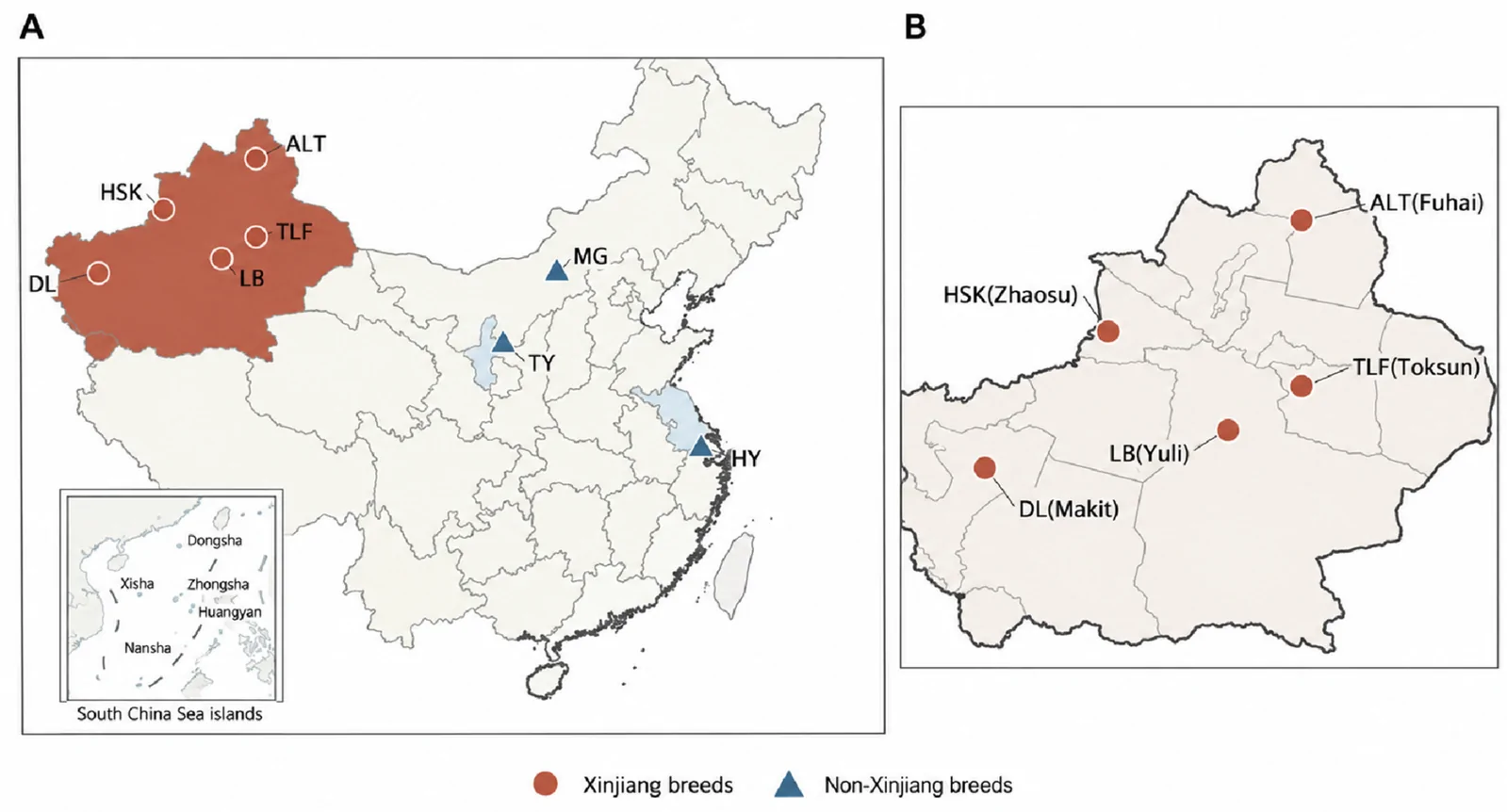
Geographic origins and sampling locations of the sheep sampled in this study. (**A**) Sampling origins of the eight sheep breeds across China, including five Xinjiang breeds (LB, HSK, DL, ALT, and TLF) and three non-Xinjiang breeds (MG, TY, and HY). (**B**) Sampling locations of the five Xinjiang breeds: Yuli (LB), Zhaosu (HSK), Makit (DL), Fuhai (ALT), and Toksun (TLF).

The five Xinjiang breeds comprised LB from Yuli, HSK from Zhaosu, DL from Makit, ALT from Fuhai and TLF from Toksun. The three non-Xinjiang breeds were MG from the Ulanhua pasture area, TY from Yanchi and HY from Wujiang. Their sampling origins are shown in Figure 1.

All animals were commercially slaughtered at local slaughter facilities in their respective sampling regions rather than at a single centralised slaughterhouse. Lambs were fasted for 24 h with free access to water and subjected to head-only electrical stunning at 1 A and 250 V for 3 s. Effective stunning was confirmed by loss of posture, apnoea and absence of the corneal reflex. Animals were exsanguinated immediately after stunning by a ventral neck cut severing both carotid arteries, and death was confirmed before further processing. No post mortem electrical stimulation was applied. Immediately after slaughter, longissimus dorsi muscles were excised at the level of the 12th–13th ribs. Visible external fat and connective tissue were removed, and the samples were subdivided according to subsequent analyses, immediately frozen in liquid nitrogen, transported under cryogenic conditions and stored at −80 °C until analysis.

### 2.2. Sample Preparation and Roasting Procedure

Frozen longissimus dorsi samples were thawed at 4 °C for 24 h. Separate portions of the same muscle sample were allocated to raw- and roasted-meat analyses. The raw portion was used for fatty acid determination, whereas an independent portion from each animal was used for volatile and aroma analyses after roasting. For roasting, the muscle from each animal was cut into 3 × 3 × 3 cm cubes and processed separately to maintain the biological replicate structure. A RATIONAL SelfCookingCenter combi oven (RATIONAL AG, Landsberg am Lech, Germany) was preheated to 180 °C before sample introduction. The meat cubes were roasted at 180 °C for 15 min, turned once and roasted for a further 10 min. The internal temperature of the meat samples at the end of roasting was not measured or recorded. No oil, salt, seasoning or other additives were used. The roasting order was randomised, and samples from different animals were never pooled. Immediately after roasting, the samples were allowed to cool to room temperature and then minced individually. The freshly prepared roasted meat was immediately subjected to HS-SPME–GC–MS and GC–O–MS analyses.

### 2.3. Fatty Acid Analysis

Fatty acids in raw lamb were determined following the method described in [11], with minor modifications. Briefly, 0.6 g of minced raw longissimus dorsi was weighed into a glass tube and spiked with 1 mL of undecanoic acid solution (5 mg/mL) as the internal standard. Total lipids were extracted with chloroform/methanol (2:1, *v*/*v*) for 24 h. The residue was re-extracted twice under the same conditions, and the three filtrates were combined and evaporated to obtain the lipid extract.

The extracted lipids were saponified with 2 mL of 0.5 mol/L methanolic NaOH until no visible lipid droplets remained. After cooling to room temperature, 2 mL of 25% boron trifluoride in methanol was added, and fatty acid methyl esters (FAMEs) were prepared at 60 °C for 30 min. After cooling, 2 mL of n-hexane and 2 mL of saturated NaCl solution were added. The mixture was vortexed and allowed to separate, and the upper organic phase was collected and centrifuged at 12,000 rpm for 15 min. The resulting supernatant was subjected to GC–FID analysis. FAMEs were analysed using a GC-2010 Plus gas chromatograph equipped with a flame ionisation detector and an AOC-20i autosampler (Shimadzu Corporation, Kyoto, Japan). Separation was achieved on an RTX-WAX capillary column (30 m × 0.32 mm i.d., 0.50 μm film thickness; Restek Corporation, Bellefonte, PA, USA). The injection volume was 0.8 μL, with a split ratio of 16:1, and the carrier-gas flow rate was 3.0 mL/min. The oven temperature was initially maintained at 120 °C for 5 min, increased to 190 °C at 10 °C/min and held for 1 min, then increased to 220 °C at 2 °C/min and held for 22 min. The injector and detector temperatures were 250 and 260 °C, respectively. Individual fatty acids were identified by comparing retention times with those of authentic FAME standards analysed under the same chromatographic conditions. Quantification was performed using undecanoic acid as the internal standard, and fatty acid contents were expressed as mg/100 g raw meat. Total saturated fatty acids (ΣSFAs), monounsaturated fatty acids (ΣMUFAs) and polyunsaturated fatty acids (ΣPUFAs) were calculated as the sums of all quantified fatty acids belonging to the corresponding classes. The PUFA/SFA ratio was calculated as ΣPUFA/ΣSFA.

### 2.4. Volatile Compound Analysis by HS-SPME–GC–MS

Volatile compounds in roasted lamb were analysed by headspace solid-phase microextraction coupled with gas chromatography–mass spectrometry (HS-SPME–GC–MS). Freshly roasted samples prepared as described in Section 2.2 were cooled to room temperature and minced. An accurately weighed portion (3.0 g) was transferred to a headspace vial, followed by the addition of 3 mL of 20% (*w*/*v*) NaCl solution and 3 μL of 2-methyl-3-heptanone solution (82 μg/mL) as the internal standard. The vial was immediately sealed.

A 50/30 μm DVB/CAR/PDMS SPME fibre (Supelco, Bellefonte, PA, USA) was conditioned according to the manufacturer’s instructions before use. The sealed vial was equilibrated at 50 °C, and the fibre was exposed to the headspace at the same temperature for 50 min. After extraction, the fibre was immediately inserted into the GC injection port and thermally desorbed for 7 min. Chromatographic separation was performed on a DB-WAX capillary column (30 m × 0.25 mm i.d., 0.25 μm film thickness; Agilent Technologies, Santa Clara, CA, USA). Samples were introduced in splitless mode, with helium as the carrier gas at a constant flow rate of 1.0 mL/min. The injector temperature was maintained at 250 °C. The oven temperature was initially held at 40 °C for 3 min, increased to 230 °C at 10 °C/min, and held at 230 °C for 6 min. Mass spectrometric detection was performed in electron ionisation mode at 70 eV. The ion-source and transfer-line temperatures were 210 and 250 °C, respectively, and mass spectra were acquired in full-scan mode over *m*/*z* 33–450. Volatile compounds were screened against the NIST 17.0 mass-spectral library (National Institute of Standards and Technology, Gaithersburg, MD, USA), retaining matches with a score of ≥80. For 18 selected volatile compounds, experimentally determined retention indices were compared with the literature values. Nine compounds were identified with support from authentic reference standards, whereas the remaining nine were tentatively identified based on mass-spectral matching and retention-index comparison. The identification evidence for each compound is summarised in Supplementary Table S1. Volatile compounds were semi-quantified using 2-methyl-3-heptanone as the internal standard, assuming a response factor of 1, and expressed as estimated μg/kg of roasted meat (internal-standard equivalents), rather than calibration-verified absolute concentrations. For class-level summaries, each volatile compound was assigned to one mutually exclusive structural class: aldehydes, alcohols, ketones/lactones, carboxylic acids, furans, pyrazines, other *N*-heterocycles, sulfur-containing compounds, phenols or others. Pyrazines were excluded from the “other *N*-heterocycles” category, and all sulfur-containing volatiles were grouped as sulfur-containing compounds irrespective of ring structure.

### 2.5. GC–O–MS Analysis

GC–O–MS analysis was performed to characterise aroma-active compounds in roasted lamb using a Shimadzu GC–MS system equipped with an ODE-2030 olfactory detection port (Shimadzu Corporation, Kyoto, Japan). Sample preparation, HS-SPME extraction conditions, chromatographic column, and oven temperature programme were identical to those described for the GC–MS analysis. The column effluent was split between the mass spectrometer and the olfactory detection port at a ratio of 1:1.

Six trained assessors (three males and three females, aged 20–25 years) participated in the GC–O analysis. Before formal evaluation, the assessors underwent approximately one month of training (approximately 2 h/day) to become familiar with the sniffing procedure, odour descriptors, and the 0–9 odour-intensity scale following established principles for GC–O assessor training and evaluation [12,13]. Samples were assigned random three-digit identification codes, and the assessors were blinded to breed information throughout the evaluation. The order of GC–O runs was randomised, with samples from different breeds distributed across assessment sessions to minimise potential order and session effects. The GC oven temperature programme lasted approximately 28 min per run. To minimise olfactory fatigue, each assessor completed no more than three runs per day, with a rest interval of at least 30 min between successive runs. Replicate evaluations of the same animal sample were conducted in separate sessions to reduce potential sensory adaptation and recall effects. Each roasted-lamb sample was evaluated independently in three separate runs by each assessor. During each run, the assessors recorded the sniffing time, odour descriptor, and perceived odour intensity. Odour intensity was scored on a 0–9 scale, with 0 indicating no perceived odour and increasing scores representing progressively greater odour intensity. The transfer delay between the MS detector and the olfactory detection port was determined using selected odorant standards and applied to correct the recorded sniffing times. Following the general principles of temporal alignment in GC–MS/O data analysis [14], odour reports with comparable descriptors were grouped within an operational window of ±30 s after time-delay correction. This window was used solely to group assessor responses rather than to establish compound identity. An odour event was retained when at least three of the six assessors reported comparable odour descriptors within this window, consistent with the assessor-consensus approach used in previous GC–O research [15]. The corresponding compounds were subsequently assigned using corrected odour-event times, chromatographic peak information, mass-spectral matching and retention-index evidence. For each animal and aroma-active compound, intensity scores from the three replicate evaluations were first averaged within each assessor and then across the six assessors to obtain the animal-level mean sniffing intensity. Non-detections were assigned an intensity of zero. Breed-level mean intensities were subsequently calculated across the six individual animals. For the occurrence analysis, a compound was considered detected at the breed level when it was detected in at least three of the six animals. The number of breeds meeting this criterion was then calculated for each compound.

### 2.6. Statistical Analysis

Statistical analyses were performed using R software (version 4.5.2). Six animals per breed were treated as independent biological replicates. Technical replicate measurements were averaged before statistical analysis, and the individual animal was considered the experimental unit. Data are presented as mean ± standard deviation (SD), unless otherwise stated.

For fatty acid variables, normality and homogeneity of variance were assessed using the Shapiro–Wilk and Levene tests, respectively. When both assumptions were satisfied, differences among breeds were evaluated by one-way analysis of variance (ANOVA), followed by Tukey’s honestly significant difference (HSD) test. When homogeneity of variance was not satisfied, Welch’s ANOVA followed by the Games–Howell test was applied. Statistical significance was set at *p* < 0.05.

For GC–MS data, semi-quantitative volatile estimates were transformed as log(1 + concentration) before statistical analysis. Breed differences in individual volatile compounds were evaluated using one-way ANOVA followed by Tukey’s HSD test, or Welch’s ANOVA followed by the Games–Howell test, as appropriate. *p*-values across volatile compounds were adjusted using the Benjamini–Hochberg false discovery rate (FDR) procedure. Principal component analysis (PCA) was performed on mean-centred and unit-variance-scaled log-transformed data. For heatmap visualisation, log-transformed abundances were standardised to Z-scores within each compound across samples.

GC–O data were summarised using mean sniffing intensities obtained from replicate evaluations by the trained assessors and were used descriptively to compare aroma-active profiles among breeds. Relationships between fatty acid variables and selected reaction-related volatile compounds were evaluated using Pearson correlation analysis based on matched individual-level observations. Linearity was assessed by visual inspection of scatterplots for each variable pair. The influence of individual observations was examined using leave-one-out sensitivity analysis to evaluate the stability of the Pearson correlation coefficients. The variables included individual unsaturated fatty acids, ΣSFA and the PUFA/SFA ratio. Correlation coefficients were visualised using a diverging colour scale, with negative and positive correlations represented separately. Correlation *p*-values were adjusted using the Benjamini–Hochberg false discovery rate (BH-FDR) procedure. Asterisks indicate significance based on adjusted *p*-values (* adjusted *p* < 0.05, ** adjusted *p* < 0.01, and *** adjusted *p* < 0.001).

## 3. Results

### 3.1. Breed-Associated Differences in Fatty Acid Composition

Fatty acid composition varied considerably among breeds (Table 1). Palmitic acid (C16:0) and stearic acid (C18:0) were the major saturated fatty acids, whereas oleic acid (C18:1n-9) predominated within the MUFA fraction. HSK and LB generally showed lower concentrations of these major fatty acids, with total SFA of 227.07 ± 34.50 and 241.83 ± 24.38 mg/100 g and total MUFA of 104.87 ± 22.45 and 113.55 ± 20.72 mg/100 g, respectively. In contrast, DL, ALT, TLF, MG, TY and HY contained substantially higher C18:1n-9, resulting in total MUFA values ranging from 236.58 ± 32.19 to 291.84 ± 40.26 mg/100 g. Total SFA was also generally higher in these breeds, reaching 357.62 ± 45.21 mg/100 g in TY and 368.41 ± 47.35 mg/100 g in HY.

More distinct variation was observed within the PUFA fraction. Linoleic acid (C18:2n-6) was highest in DL and HY, at 118.63 ± 21.84 and 111.84 ± 20.63 mg/100 g, respectively, whereas HSK showed the lowest level (50.04 ± 16.08 mg/100 g). Arachidonic acid (C20:4n-6) followed a similar tendency, with comparatively high concentrations in HY (43.16 ± 8.42 mg/100 g) and DL (39.18 ± 7.86 mg/100 g). By contrast, α-linolenic acid (C18:3n-3) occurred at lower absolute concentrations across all breeds and was highest in HSK. Consequently, total PUFA was greatest in DL (221.86 ± 29.74 mg/100 g) and HY (207.63 ± 30.18 mg/100 g), while lower levels were observed in HSK and TLF. These differences indicate marked breed-associated variation in the pool of unsaturated lipid precursors available for thermal oxidation and provided a basis for examining whether the fatty acid patterns were reflected in the roasted volatile profiles.

### 3.2. Breed-Associated Variation in the Roasted Volatile Profile

**Figure 2:**
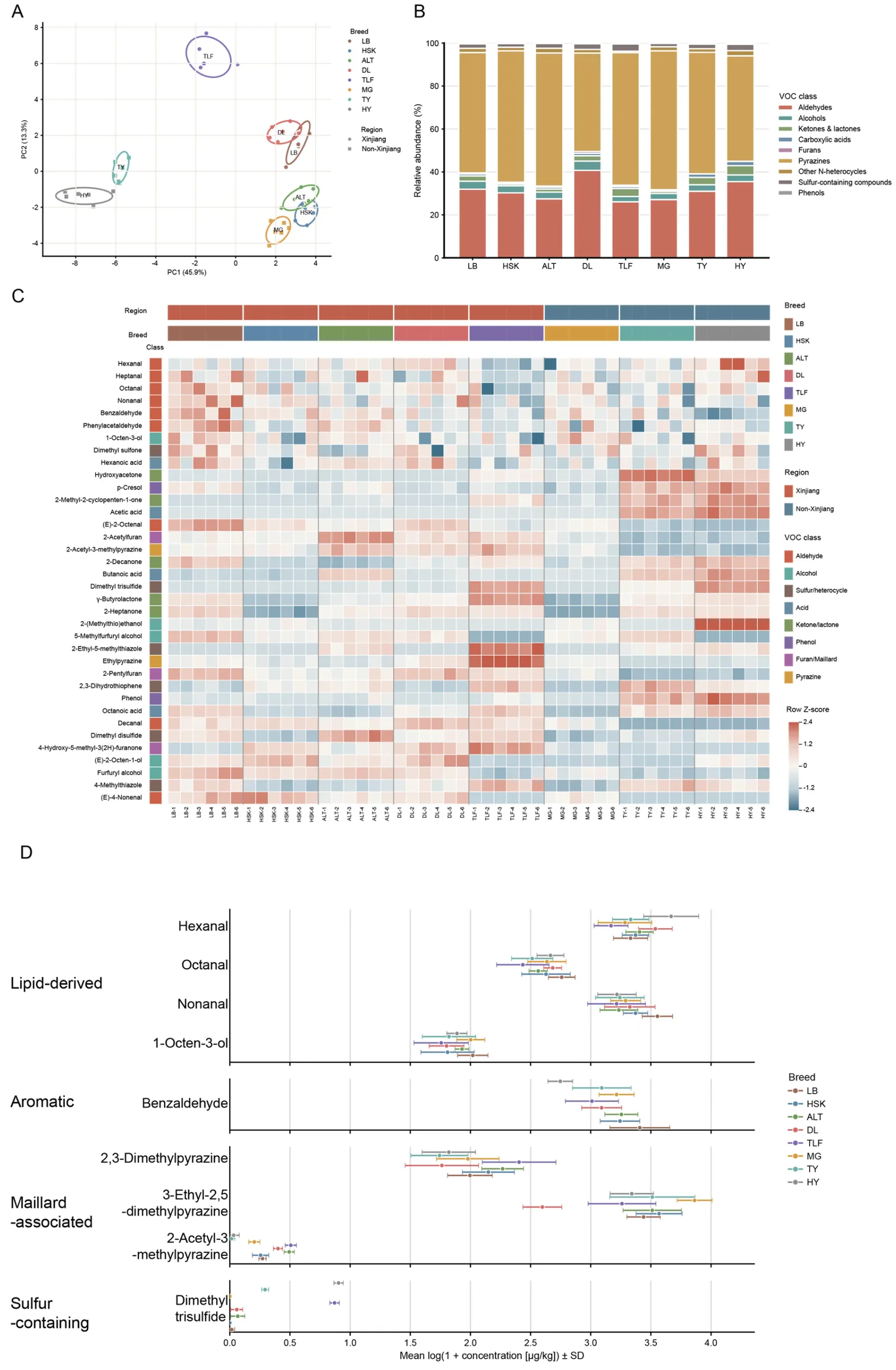
Breed-associated variation in the roasted volatile profiles of the eight sheep breeds. (**A**) Exploratory principal component analysis (PCA) of individual samples. (**B**) Relative contributions of volatile classes based on semi-quantitative GC–MS estimates. (**C**) Heatmap of representative compounds across individual samples, shown as within-compound Z-scores. (**D**) Mean log(1 + estimated concentration [μg/kg]) ± SD of selected volatile compounds.

The roasted volatile profiles showed distinct distribution patterns among the eight breeds (Figure 2A–D). PCA accounted for 59.2% of the total variation, with PC1 and PC2 explaining 45.9% and 13.3%, respectively (Figure 2A). HSK and ALT clustered closely, with MG positioned in the same positive-PC1 region, whereas LB and DL showed substantial overlap. TLF appeared separated along PC2, while TY and HY were located on the negative side of PC1. The sample distribution suggested breed-associated patterns rather than a simple Xinjiang/non-Xinjiang separation, but PCA alone does not establish statistical significance. Aldehydes and pyrazines were the dominant volatile classes across all breeds, although their relative contributions differed markedly (Figure 2B). DL showed the greatest relative contribution of aldehydes, whereas HSK, ALT, TLF and MG were characterised by larger pyrazine fractions. LB and TY showed intermediate class distributions, while HY retained a comparatively greater aldehyde contribution than most of the pyrazine-rich breeds. The remaining volatile fraction consisted of smaller contributions from alcohols, ketones and lactones, carboxylic acids, furans, other N-heterocycles, sulfur-containing compounds, phenols and other minor volatiles. These class-level differences were also reflected at the individual-compound level. The sample-level heatmap showed distinct breed-associated patterns among representative aldehydes, alcohols, pyrazines and other volatile compounds, while retaining appreciable variation among individuals within the same breed (Figure 2C).

The semi-quantitatively estimated abundances of selected compounds further illustrated these breed-associated patterns (Figure 2D). Hexanal showed relatively high mean abundances in HY and DL, whereas LB showed comparatively high levels of octanal, nonanal and 1-octen-3-ol. Benzaldehyde was also relatively abundant in LB and lower in HY. Among the Maillard-associated compounds, TLF showed comparatively high mean levels of 2,3-dimethylpyrazine and 2-acetyl-3-methylpyrazine, whereas 3-ethyl-2,5-dimethylpyrazine was more abundant in MG and comparatively low in DL. Dimethyl trisulfide was particularly prominent in HY and TLF relative to the other breeds. Overall, breed-associated variation in the roasted volatilome reflected shifts in the relative abundance of lipid-derived, aromatic, Maillard-associated and sulfur-containing compounds rather than the occurrence of fundamentally different volatile classes.

### 3.3. Shared and Breed-Specific Aroma-Active Compounds Detected by GC–O–MS

**Figure 3:**
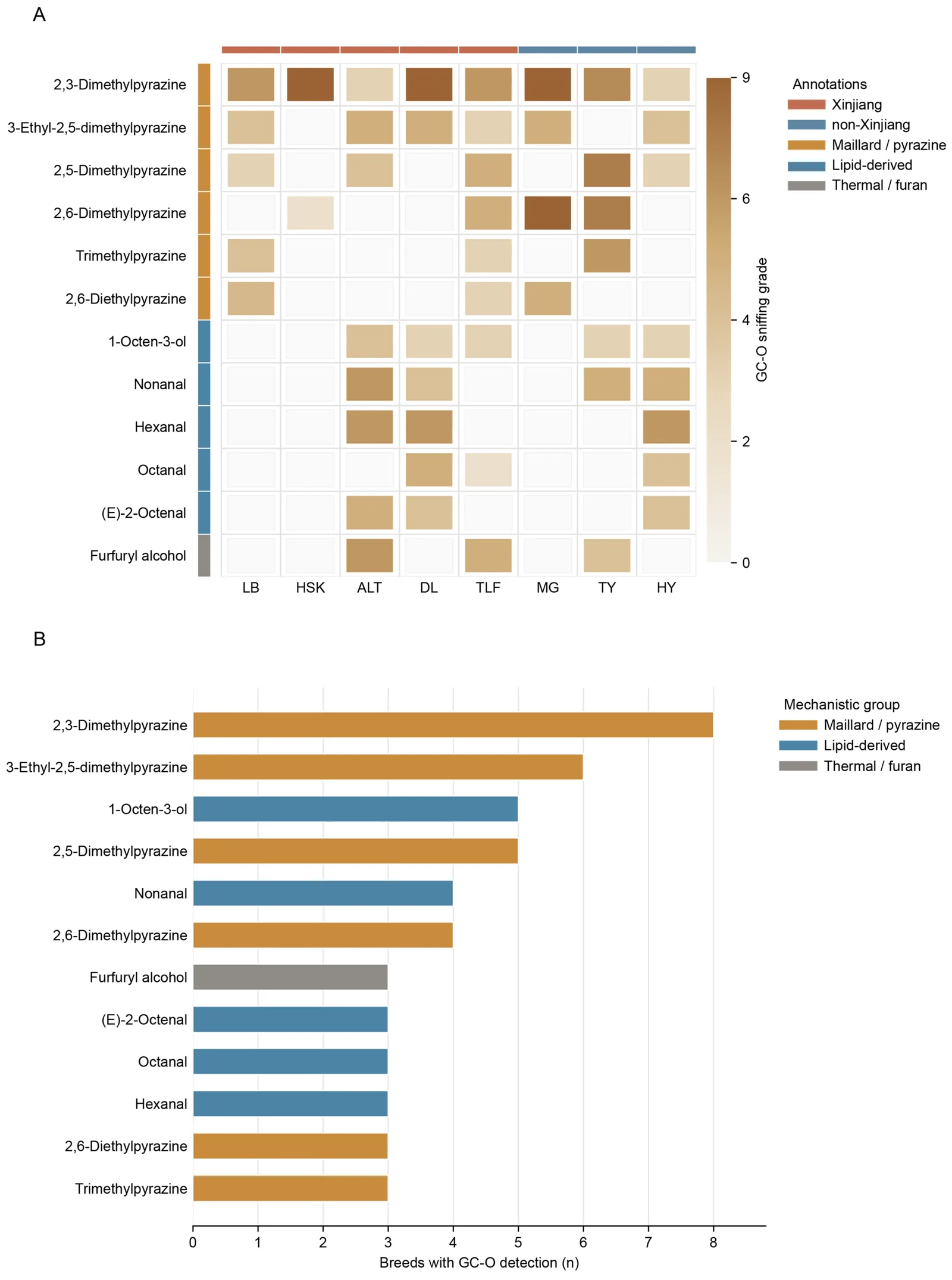
GC–O detection and sniffing grades of aroma-active compounds across eight sheep breeds. (**A**) Heatmap showing breed-level mean GC–O sniffing grades (0–9), calculated from six individual animals per breed, with non-detections assigned zero. (**B**) Number of breeds in which each aroma-active compound was detected in at least three of the six animals. Sniffing grades represent olfactory-port responses rather than overall sensory intensity of roasted meat.

GC–O analysis showed that the eight breeds shared a common set of aroma-active compounds but differed in both their occurrence patterns and sniffing grades (Figure 3A,B). Pyrazines were the most recurrent odorant group. 2,3-Dimethylpyrazine was detected in all breeds and was associated mainly with roasted, nutty, cereal-like and cocoa-like notes. Its highest sniffing grades were observed in HSK, DL and MG. Other alkylpyrazines, including 3-ethyl-2,5-dimethylpyrazine, 2,5-dimethylpyrazine and 2,6-dimethylpyrazine, contributed additional roasted, nutty and cocoa-like characteristics, but their distribution differed markedly among breeds.

Distinct GC–O aroma profiles were evident from the combination of detected aroma-active compounds. HSK and MG were characterised by relatively simple but intense pyrazine-dominated profiles, with high sniffing grades for 2,3-dimethylpyrazine and, in MG, 2,6-dimethylpyrazine, indicating a pronounced roasted and nutty character. LB also showed a predominantly pyrazine-based profile, but with a broader contribution from several alkylpyrazines, suggesting a more complex roasted, cereal-like and cocoa-like aroma. TLF contained the widest combination of pyrazines together with furfuryl alcohol, giving a profile characterised by roasted, nutty and sweet/caramel-like notes.

In contrast, ALT and DL showed a greater contribution from lipid-derived odorants. ALT contained hexanal, nonanal, (*E*)-2-octenal and 1-octen-3-ol at moderate-to-high sniffing grades, corresponding to green, grassy, fatty, citrus-like and mushroom-like notes, together with a smaller pyrazine component. DL showed a similar mixed profile, with strong hexanal and moderate nonanal, octanal and (*E*)-2-octenal, superimposed on high 2,3-dimethylpyrazine, resulting in a combination of roasted/nutty and green/fatty/citrus-like odour characteristics.

TY displayed one of the broadest pyrazine profiles, particularly for 2,5-dimethylpyrazine, 2,6-dimethylpyrazine and trimethylpyrazine, while nonanal, 1-octen-3-ol and furfuryl alcohol added fatty, mushroom-like and sweet/caramel-like notes. HY showed a comparatively stronger lipid-derived odour profile, with high sniffing grades for hexanal and nonanal together with detectable octanal, (*E*)-2-octenal and 1-octen-3-ol, suggesting a greater contribution from green, fatty, citrus-like and mushroom-like odour characteristics than from the roasted pyrazine fraction.

Overall, HSK, LB and MG were more strongly associated with roasted and nutty pyrazine-derived odour characteristics, ALT and DL showed a mixed roasted–lipid-derived profile, TLF combined roasted/nutty and sweet/caramel-like attributes, TY was characterised by a broad pyrazine-dominated aroma profile, and HY showed a stronger contribution from lipid-derived green/fatty odorants.

### 3.4. Relationships Between Fatty Acid Composition and Reaction-Related Volatile Profiles

**Figure 4:**
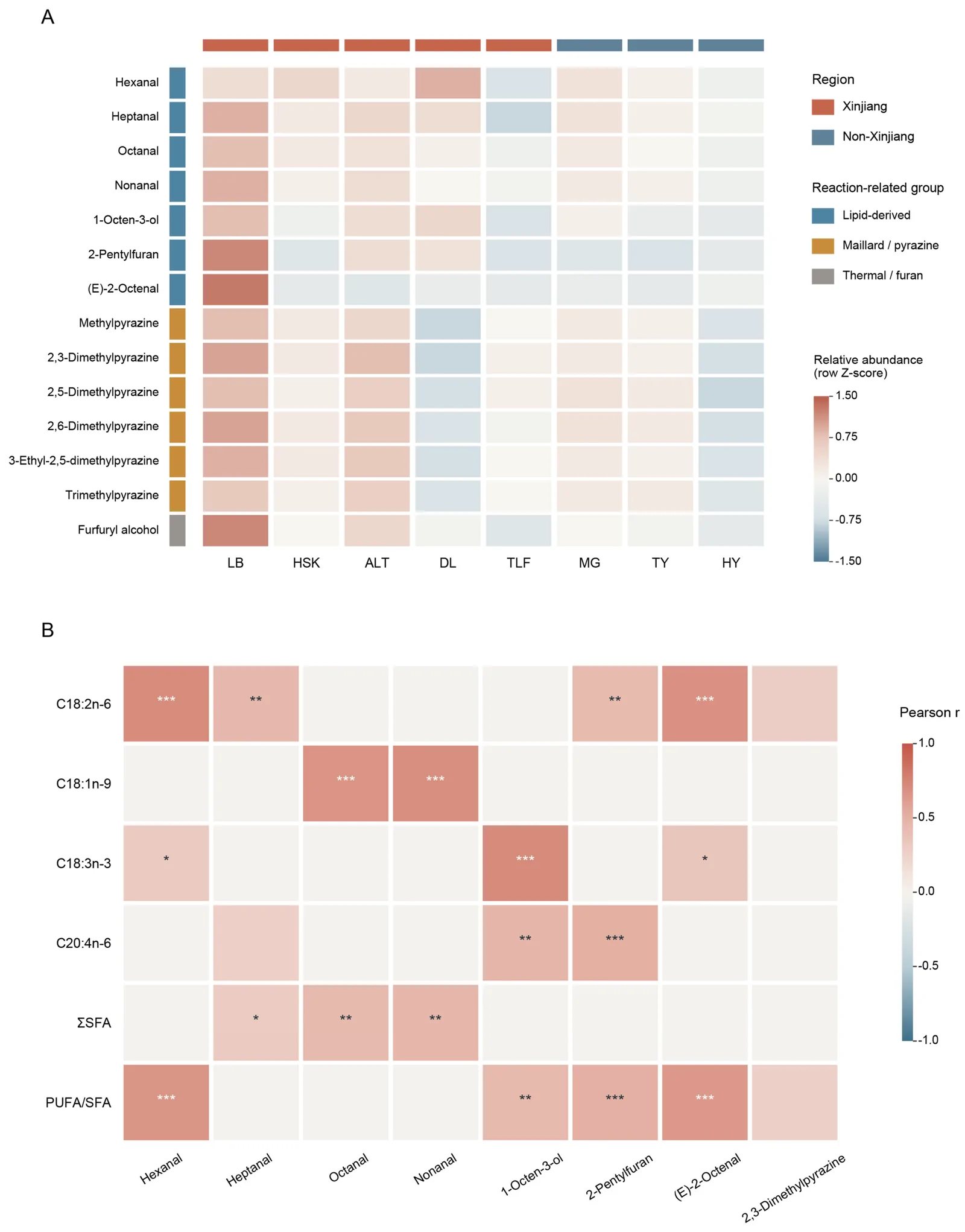
Relationships between fatty acid composition and selected roasted volatile compounds. (**A**) Row-standardised heatmap (Z-scores) of semi-quantitative volatile profiles across the eight breeds. (**B**) Pearson correlation matrix based on matched individual-animal observations. Asterisks indicate significance based on Benjamini–Hochberg-adjusted *p*-values (* adjusted *p* < 0.05, ** adjusted *p* < 0.01 and *** adjusted *p* < 0.001). Asterisk colours are used for contrast and do not indicate different significance levels.

The reaction-related volatile profiles varied among breed-origin groups (Figure 4A). LB exhibited relatively high abundances across a broad range of lipid-derived and Maillard-associated compounds, including several aldehydes, 1-octen-3-ol, 2-pentylfuran and alkylpyrazines. DL showed a more lipid-oriented profile, with relatively high levels of hexanal, 1-octen-3-ol and 2-pentylfuran but lower abundances of most selected pyrazines. ALT, in contrast, showed comparatively higher levels of several alkylpyrazines. The remaining breeds displayed more compound-specific patterns, and no consistent separation between Xinjiang and non-Xinjiang breeds was evident. The fatty acid–volatile correlation matrix showed that BH-FDR-significant associations were concentrated mainly among lipid-related variables and lipid-derived volatiles (Figure 4B). C18:2n-6 was significantly positively associated with hexanal, heptanal, 2-pentylfuran and (*E*)-2-octenal, whereas C18:1n-9 showed significant positive associations with octanal and nonanal. C18:3n-3 was significantly associated with hexanal, 1-octen-3-ol and (*E*)-2-octenal, while C20:4n-6 was positively associated with 1-octen-3-ol and 2-pentylfuran. ΣSFA showed significant positive associations with heptanal, octanal and nonanal. The PUFA/SFA ratio was also positively associated with several lipid-derived volatiles, including hexanal, 1-octen-3-ol, 2-pentylfuran and (*E*)-2-octenal. In contrast, no significant association was observed between the fatty acid variables and 2,3-dimethylpyrazine.

Overall, the significant fatty acid–volatile associations were concentrated within the lipid-derived component of the roasted volatilome, whereas the pyrazine fraction showed comparatively little association with fatty acid composition.

## 4. Discussion

### 4.1. Fatty Acid Precursor Background and Lipid-Derived Volatile Formation

Variation in muscle fatty acids among breeds is expected to reflect both genetic background and production conditions. In lamb, total lipid deposition strongly affects the absolute amounts of SFA and MUFA, whereas forage availability and feeding strategy have a greater influence on the PUFA profile. Recent reviews consistently show that pasture-based systems generally increase the deposition of C18:3n-3 and other n-3 PUFAs while reducing the n-6/n-3 ratio relative to concentrate-based feeding [16,17]. These studies indicate that feeding conditions may contribute to the variation in fatty acid composition observed among the sampled breed-origin groups. Experimental evidence also supports this response: lambs grazing sainfoin pasture showed higher PUFAs, particularly n-3 PUFA, than indoor-fed lambs, together with improved oxidative stability of the muscle [18]. Similar differences between grazing and indoor systems have recently been reported in Duhu lamb, where grazing increased n-3 PUFA and reduced SFA deposition [19]. The higher C18:2n-6, C20:4n-6 and total PUFA contents in DL and HY are more relevant to the subsequent flavour analysis because unsaturated fatty acids provide an important substrate pool for thermal oxidation. Recent work on roasted lamb chops showed that oxidation of oleic, linoleic and linolenic acids was closely associated with the formation of characteristic volatile compounds, particularly aldehydes and related lipid-derived products [20].

However, a higher PUFA content should be interpreted as greater precursor availability rather than a direct prediction of stronger aroma. Pasture-derived antioxidants can simultaneously improve oxidative stability and limit excessive lipid oxidation, even when muscle PUFA is increased [16,18]. Thus, the fatty-acid differences observed among breeds provide a biochemical basis for variation in lipid-derived flavour formation, but their contribution ultimately depends on the extent of oxidation during roasting.

### 4.2. Shared Aroma-Active Backbone and Breed-Associated Aroma Variation

Similar breed-associated variation in lamb volatile profiles has been reported previously [21,22]. The predominance of aldehydes and pyrazines observed here is consistent with the major chemical routes involved in roasted-lamb aroma formation. Lipid oxidation generates aldehydes, alcohols and ketones, whereas Maillard reactions contribute nitrogen- and sulfur-containing heterocyclic compounds; interactions between these pathways further modify the final aroma profile [23]. The relatively aldehyde-rich profile of DL coincided with comparatively high abundances of several oxidation-sensitive fatty acids. Hexanal, octanal, nonanal and 1-octen-3-ol are frequently reported lipid-derived volatiles in roasted lamb, and breed-related differences in PUFA composition have previously been accompanied by variation in these compounds [22]. Precursor abundance alone, however, does not determine volatile formation. Feeding background, antioxidant status and thermal conditions can all influence lipid oxidation. For example, pasture feeding has been shown to modify both fatty acid composition and volatile profiles in Sunit sheep [24]. The comparatively large pyrazine fraction observed in HSK, ALT and MG, together with the abundance of individual alkylpyrazines in TLF and MG, is consistent with a greater relative contribution of Maillard chemistry. Pyrazines are characteristic products of thermal amino–carbonyl reactions and are widely detected in roasted and grilled lamb products [25]. Sulfur-containing compounds provided an additional source of differentiation, particularly the relatively high dimethyl trisulfide abundance in HY and TLF. Despite their lower concentrations, such compounds can remain aroma-relevant because of their low odour thresholds [26].

Overall, the GC–MS profiles differed mainly in the relative contribution of lipid-derived aldehydes, Maillard-derived pyrazines and sulfur-containing compounds rather than in the presence of entirely different volatile systems.

### 4.3. Relative Contributions of Lipid Oxidation and Maillard Chemistry

The recurrent detection of 2,3-dimethylpyrazine and related alkylpyrazines across breeds indicates that roasted and nutty notes represent a common component of roasted-lamb aroma. Pyrazines are typical Maillard-derived odorants generated during high-temperature processing, and several have been identified as key aroma compounds in roasted mutton by GC–O and aroma-recombination approaches [27,28]. The stronger pyrazine-associated profiles of HSK, LB, MG and TY therefore suggest differences mainly in the intensity and diversity of Maillard-derived odorants rather than in the presence of an entirely different aroma system. In contrast, ALT, DL and HY showed a greater contribution from lipid-derived odorants. Hexanal, octanal, nonanal, (*E*)-2-octenal and 1-octen-3-ol are repeatedly reported as important odorants in roasted mutton, contributing green, fatty, citrus-like and mushroom-like characteristics. Aroma-recombination and omission experiments have confirmed hexanal, octanal, nonanal and 1-octen-3-ol among the compounds with substantial effects on roasted-mutton aroma [27,29]. The broader combination of pyrazines and furfuryl alcohol in TLF is consistent with a more complex thermally generated aroma profile, combining roasted/nutty characteristics with sweet or caramel-like notes. Differences in roasting conditions have been shown to markedly alter both pyrazine formation and the balance between roasted, fatty and sweet aroma attributes in lamb [30]. The breed-related differences observed here are therefore better interpreted as changes in the relative contribution of several odour-active groups.

Recent GC–O studies similarly show that roasted-mutton aroma is usually determined by a limited subset of aldehydes, alcohols, pyrazines and sulfur-containing compounds rather than by the total volatile pool [8,31]. GC–O sniffing grades provide semi-quantitative information on the perceived intensity of individual aroma-active compounds, complementing the volatile abundance data obtained by GC–MS [32]. Importantly, chemical abundance and olfactory contribution were not always aligned. The GC–MS data indicated a relatively large pyrazine contribution in several breeds, whereas GC–O showed that lipid-derived aldehydes and alcohols could make prominent olfactory contributions even when they were not the dominant fraction of the total volatilome. ALT provides a clear example: its GC–MS profile contained a substantial pyrazine component, while its GC–O profile also showed pronounced contributions from hexanal, nonanal, (*E*)-2-octenal and 1-octen-3-ol. Conversely, 2,3-dimethylpyrazine was repeatedly detected by GC–O across all breeds despite variation in its chemical abundance. This divergence illustrates the complementary information provided by GC–MS and GC–O and supports the distinction between volatile abundance and olfactory relevance.

### 4.4. Fatty Acid–Volatile Associations and Reaction Pathways

The associations between fatty acids and lipid-derived volatiles were consistent with established relationships between unsaturated lipid oxidation and volatile formation during lamb roasting. C18:2n-6 was positively associated with hexanal and (*E*)-2-octenal, whereas C18:1n-9 showed stronger associations with octanal and nonanal. Similar relationships have been reported during lamb heating, where oxidation of unsaturated fatty acids and phospholipids contributed to straight-chain aldehydes and other lipid-derived volatiles [18,33]. Comparable associations between PUFA composition and hexanal, heptanal, octanal and nonanal have also been observed among lamb breeds [22].

The positive associations of the PUFA/SFA ratio with several lipid-derived volatiles are consistent with the greater susceptibility of unsaturated lipid pools to thermal oxidation. Nevertheless, fatty acid availability represents only one component of volatile formation, which is also influenced by oxidative stability, thermal exposure and interactions among reaction intermediates [30]. In contrast, the comparatively weak associations between fatty acid variables and 2,3-dimethylpyrazine are consistent with the predominant formation of pyrazines through Maillard chemistry rather than direct lipid oxidation. Lipid-derived carbonyls may nevertheless interact with Maillard intermediates during heating, and the two pathways should therefore not be considered completely independent [34,35].

Significant fatty acid–volatile associations were concentrated in lipid-derived compounds, particularly aldehydes, whereas 2,3-dimethylpyrazine showed no significant correlation with the fatty acids examined. These results highlight the contribution of lipid-derived volatiles to the differences observed among the sampled groups, alongside the common pyrazine-related aroma features detected by GC–O.

Under a common roasting programme, differences in fatty acid composition and roasted volatile profiles were observed among the eight breed-origin groups. However, each breed was sampled from a single farm and slaughtered locally, so breed, geographical origin and production conditions could not be disentangled. Meat core temperatures were not recorded, and fatty acid–volatile correlations may partly reflect between-group differences rather than direct precursor–product relationships. These findings therefore describe breed-associated patterns under the sampled conditions, warranting further validation across multiple farms and under more controlled processing conditions.

## 5. Conclusions

The eight sheep breeds shared a common roasted aroma-active background dominated by alkylpyrazines, particularly 2,3-dimethylpyrazine, while breed-associated differences were mainly reflected in the relative contributions of lipid-derived and Maillard-derived odorants. Some breeds showed more pyrazine-rich roasted/nutty profiles, whereas others showed greater contributions from lipid-derived aldehydes and alcohols. Unsaturated fatty acids were more closely associated with lipid-derived volatiles than with pyrazines, linking variation in lipid composition with part of the roasted volatilome. GC–O–MS provided complementary information on olfactory relevance that was not captured by volatile abundance alone. Overall, roasted aroma differences among breeds were characterised primarily by shifts in the balance between lipid oxidation and Maillard-derived compounds against a broadly shared aroma-active background. The animals originated from different geographical regions and routine production systems; therefore, these differences represent breed-associated patterns rather than independent genetic effects.

## Figures and Tables

**Table 1:** Major fatty acid contents in the raw longissimus dorsi muscle of eight sheep breeds (mg/100 g raw meat).

Fatty Acid	LB	HSK	ALT	DL	TLF	MG	TY	HY
Palmitic acid (C16:0)	119.68 ± 10.86 ^bc^	116.11 ± 15.93 ^c^	173.62 ± 23.48 ^a^	167.84 ± 27.63 ^a^	158.93 ± 20.76 ^ab^	169.45 ± 23.61 ^a^	181.76 ± 25.87 ^a^	186.93 ± 27.14 ^a^
Stearic acid (C18:0)	106.30 ± 13.17 ^bc^	89.23 ± 16.74 ^c^	132.48 ± 18.27 ^ab^	126.92 ± 21.46 ^ab^	124.67 ± 16.84 ^ab^	136.27 ± 19.63 ^ab^	141.83 ± 20.57 ^a^	146.28 ± 21.86 ^a^
Oleic acid (C18:1n-9)	88.77 ± 16.66 ^b^	80.61 ± 19.01 ^b^	208.36 ± 29.47 ^a^	228.47 ± 35.62 ^a^	191.42 ± 26.83 ^a^	216.73 ± 31.26 ^a^	231.58 ± 33.74 ^a^	226.71 ± 32.86 ^a^
Linoleic acid (C18:2n-6)	88.41 ± 24.44 ^abc^	50.04 ± 16.08 ^d^	83.74 ± 15.62 ^bcd^	118.63 ± 21.84 ^a^	74.86 ± 14.71 ^cd^	79.42 ± 15.83 ^bcd^	89.63 ± 17.46 ^abc^	111.84 ± 20.63 ^ab^
α-Linolenic acid (C18:3n-3)	7.16 ± 2.05 ^b^	12.85 ± 4.57 ^a^	8.61 ± 1.94 ^ab^	9.24 ± 2.36 ^ab^	7.38 ± 1.72 ^b^	9.71 ± 2.18 ^ab^	7.16 ± 1.75 ^b^	6.27 ± 2.31 ^b^
Arachidonic acid (C20:4n-6)	34.90 ± 10.54 ^abc^	25.15 ± 9.37 ^bc^	27.46 ± 5.82 ^bc^	39.18 ± 7.86 ^ab^	23.71 ± 5.34 ^c^	29.86 ± 6.48 ^abc^	30.72 ± 6.91 ^abc^	43.16 ± 8.42 ^a^
ΣSFA	241.83 ± 24.38 ^bc^	227.07 ± 34.50 ^c^	342.18 ± 41.36 ^a^	323.75 ± 44.68 ^a^	313.84 ± 37.42 ^ab^	337.29 ± 42.16 ^a^	357.62 ± 45.21 ^a^	368.41 ± 47.35 ^a^
ΣMUFA	113.55 ± 20.72 ^b^	104.87 ± 22.45 ^b^	263.92 ± 35.41 ^a^	289.41 ± 39.27 ^a^	236.58 ± 32.19 ^a^	272.14 ± 37.63 ^a^	291.84 ± 40.26 ^a^	283.52 ± 38.47 ^a^
ΣPUFA	158.29 ± 42.47 ^bc^	123.97 ± 40.84 ^c^	157.43 ± 25.38 ^bc^	221.86 ± 29.74 ^a^	139.52 ± 23.46 ^c^	151.68 ± 24.71 ^bc^	162.47 ± 26.53 ^bc^	207.63 ± 30.18 ^ab^

Values are expressed as mean ± standard deviation (SD; n = 6 independent animals per breed). Within a row, means without a shared superscript letter differ significantly (*p* < 0.05). SFA, saturated fatty acid; MUFA, monounsaturated fatty acid; PUFA, polyunsaturated fatty acid. ΣSFA, ΣMUFA and ΣPUFA denote sums of the quantified fatty acids in their respective classes.

## Data Availability

The original data presented in this study are openly available in Figshare at https://doi.org/10.6084/m9.figshare.29546306.

## Supplementary Materials

The following supporting information can be downloaded at: https://www.mdpi.com/article/10.3390/foods15213873/s1, Table S1: Experimental and literature retention indices and identification methods for selected volatile compounds in roasted lamb.

## References

[1] Xiang C., Li S., Zhang D., Huang C., Zhao Y., Zheng X., Wang Z., Chen L. (2023). Characterization and discrimination of the flavor profiles of chinese indigenous sheep breeds via electronic sensory, smart instruments and chemometrics. J. Food Compos. Anal..

[2] Wang F., Wang H., Liang Z., Liu J., Yang C., Zhai H., Chen A., Lu Z., Gao Y., Ding X. (2024). Characterization of flavor compounds in chinese indigenous sheep breeds using gas chromatography-ion mobility spectrometry and chemometrics. Foods.

[3] Xu Y., Yang Q., Zheng X., Yang H., Schroyen M., Zhang D., Hou C. (2025). Identification of volatile flavor substances in four key muscle portions of five tibetan sheep breeds. J. Sci. Food Agric..

[4] Yang D., Gao S., Yuan H., Zhao W., Luo Y., Luo R., Hou Y. (2024). Formation mechanisms of odor substances in mutton and deodorization methods: A review. Food Sci..

[5] Yang Z., Cui Z., Zhang M., Sun L., Zhao L., Su L., Jin Y. (2025). Analysis of the effects of hydroxyl radicals on the volatile flavor composition and lipid profile of sheep meat based on hs-spme-gc-ms and uplc-ms/ms studies. Food Chem..

[6] Wu Y., Qi X., Li Q., Zhang L., Ma Y. (2026). Linking aroma precursors and volatile compounds to authenticate dongxiang tribute sheep meat. Food Chem. X.

[7] Wu T., Wang P., Zhang Y., Zhan P., Zhao Y., Tian H., He W. (2023). Identification of muttony-related compounds in cooked mutton tallows and their flavor intensities subjected to phenolic extract from thyme (*thymus vulgaris* l.). Food Chem..

[8] Xie Y., Zhang Y., Qi W., Zhang Y., Zhou G., Liu D. (2026). Characterization of key aroma-active components in roast mutton: Integration of gc-ims, gc-ms, and aroma recombination. Food Chem. X.

[9] Zhang Y., Wang Y., Liu H., Xu D., Liu D. (2025). Development of a fiber derivatization method for the analysis of 18 carbonyl compounds in roasted lamb using hs-spme/gc-tq/ms. Food Chem..

[10] (2016). Animal welfare management—General requirements and guidance for organizations in the food supply chain.

[11] Chen B., Zeng M., Shen X., Liu W. (2026). Flavour signatures of duolang lamb from arid-desert and temperate regions: Precursor chemistry, aroma validation and metabolomic links. Food Res. Int..

[12] Delahunty C.M., Eyres G., Dufour J.-P. (2006). Gas chromatography-olfactometry. J. Sep. Sci..

[13] Brattoli M., Cisternino E., Dambruoso P.R., De Gennaro G., Giungato P., Mazzone A., Palmisani J., Tutino M. (2013). Gas chromatography analysis with olfactometric detection (GC-O) as a useful methodology for chemical characterization of odorous compounds. Sensors.

[14] Gerretzen J., Buydens L.M.C., Tromp-van den Beukel A.O., Koussissi E., Brouwer E.R., Jansen J.J., Szymanska E. (2015). A novel approach for analyzing gas chromatography-mass spectrometry/olfactometry data. Chemom. Intell. Lab. Syst..

[15] Zhao Y., Chen Y., Gao M., Wu L., Wang Y. (2023). Comparative investigation of key aroma terpenoids of *Litsea cubeba* essential oil by sensory, chromatographic, spectral and molecular studies. LWT.

[16] Kearns M., Ponnampalam E.N., Jacquier J.C., Grasso S., Boland T.M., Sheridan H., Monahan F.J. (2023). Can botanically-diverse pastures positively impact the nutritional and antioxidant composition of ruminant meat?—Invited review. Meat Sci..

[17] Alvarez-Rodriguez J., Urrutia O., Lobon S., Ripoll G., Bertolin J.R., Joy M. (2022). Insights into the role of major bioactive dietary nutrients in lamb meat quality: A review. J. Anim. Sci. Biotechnol..

[18] Li Z., Peng C., Wang H., Liu X. (2024). Improved muscle fatty acid composition and oxidative stability in lambs grazing on sainfoin pasture. Vet. Anim. Sci..

[19] Jia W., Yang J., Zhang B., Sun S., Dou X., Ren G., Wang Y. (2024). Evaluation of meat quality in duhu hybrid lambs reared in different conditions. Foods.

[20] Li K., Wang X., Liu Y., Wang P., Wang J., Tian H., Xie J. (2024). Effects of cooking methods on fatty acid composition and volatile substances in oven-roasted lamb chops. Food Sci..

[21] Zhang C., Zhang H., Liu M., Zhao X., Luo H. (2020). Effect of breed on the volatile compound precursors and odor profile attributes of lamb meat. Foods.

[22] Wang J., Song Y., Zhao L., Yang P., Huang F., Wu Y., Zhang C. (2024). Characterization and discrimination of lamb with different breeds and muscle types using precursors and volatile compounds. Food Chem. X.

[23] Sohail A., Al-Dalali S., Wang J., Xie J., Shakoor A., Asimi S., Shah H., Patil P. (2022). Aroma compounds identified in cooked meat: A review. Food Res. Int..

[24] Yang Z., Liu C., Dou L., Chen X., Zhao L., Su L., Jin Y. (2022). Effects of feeding regimes and postmortem aging on meat quality, fatty acid composition, and volatile flavor of longissimus thoracis muscle in sunit sheep. Animals.

[25] Shen C., Cai Y., Wu X., Gai S., Wang B., Liu D. (2023). Characterization of selected commercially available grilled lamb shashliks based on flavor profiles using gc-ms, gc x gc-tof-ms, gc-ims, e-nose and e-tongue combined with chemometrics. Food Chem..

[26] Li J., Tang C., Yang Y., Hu Y., Zhao Q., Ma Q., Yue X., Li F., Zhang J. (2023). Characterization of meat quality traits, fatty acids and volatile compounds in hu and tan sheep. Front. Nutr..

[27] Liu H., Hui T., Fang F., Li S., Wang Z., Zhang D. (2022). The formation of key aroma compounds in roasted mutton during the traditional charcoal process. Meat Sci..

[28] Liang B., Li J., Zhao S., Pan X., Zhang Y., Gao P., Li P., Xing J., Suleman R., Gong H. (2024). Comprehensive analysis of key aroma compounds enhanced by tamarix ramosissima ledeb in mutton roasted by air-frying roast technology by means of safe-gc-o-ms and lipidomics. Food Chem. X.

[29] Liu H., Hui T., Fang F., Ma Q., Li S., Zhang D., Wang Z. (2021). Characterization and discrimination of key aroma compounds in pre- and postrigor roasted mutton by gc-o-ms, gc e-nose and aroma recombination experiments. Foods.

[30] Li K., Wang X., Liu Y., He W., Zhan P., Tian H., Xie J. (2025). Influence of different cooking methods on quality characteristics, nutritional value, and volatile compounds of roasted lamb chops. J. Food Compos. Anal..

[31] Liu H., Li J., Zhang D., Hamid N., Liu D., Hua W., Du C., Ma Q., Gong H. (2023). The effect of thermal times of circulating non-fried roast technique on the formation of (non)volatile compounds in roasted mutton by multi-chromatography techniques and heat transfer analysis. Food Res. Int..

[32] Mahmoud M.A.A., Zhang Y. (2024). Enhancing odor analysis with gas chromatography-olfactometry (gc-o): Recent breakthroughs and challenges. J. Agric. Food Chem..

[33] Cheng K., Liu T., Yang C., Yang H., Liu D. (2024). Relationship between phospholipid molecules species and volatile compounds in grilled lambs during the heating process. Food Chem. X.

[34] Hua W., Liu H., Fei Q., Wu J., Wei X., Ebaid M., Tao R., Liu D., Gai S. (2025). Formation of volatile compounds in mutton during air fryer roasted mutton by means of gc-ms and gc-ims. Food Res. Int..

[35] Wei X., Nie R., Brunton N.P., Dunne P.G., Monahan F.J., Zhang C., Wang Z., Zhang D. (2025). Formation of volatile flavours and heterocyclic aromatic amines in roasted lamb leg. LWT.

