# Supplementary material for: Breed-Associated Roasted Aroma Signatures Across Eight Chinese Sheep Breeds: Integrating Fatty Acid Composition, Volatile Profiles, and Aroma-Active Compounds
Source: Foods. 2026 Oct 29;15(21):3873. doi: 10.3390/foods15213873 (PMC13762637; doi:10.3390/foods15213873)
Supplement: Supplementary file 1 [file foods-15-03873-s001.zip › foods-4593012-supplementary.pdf]

Table S1. Experimental and literature retention indices and identification methods for selected volatile compounds in roasted lamb.

Compound	CAS	RI exp.	RI lit.	Identification
Hexanal	66-25-1	1068	1068	MS, RI, O, S
Heptanal	111-71-7	1160	1160	MS, RI, S
Octanal	124-13-0	1277	1293	MS, RI, O, S
Nonanal	124-19-6	1367	1367	MS, RI, O, S
Benzaldehyde	100-52-7	1528	1537	MS, RI, S
1-Octen-3-ol	3391-86-4	1458	1462	MS, RI, O, S
(E)-2-Octenal	2548-87-0	1392	1392	MS, RI, O, S
2-Pentylfuran	3777-69-3	1228	1228	MS, RI, S
2,3-Dimethylpyrazine	5910-89-4	1342	1357	MS, RI, O
2,5-Dimethylpyrazine	123-32-0	1323	1323	MS, RI, O
2,6-Dimethylpyrazine	108-50-9	1331	1337	MS, RI, O
3-Ethyl-2,5-dimethylpyrazine	13360-65-1	1447	1450	MS, RI, O
2-Acetyl-3-methylpyrazine	23787-80-6	1628	1640	MS, RI
Methylpyrazine	109-08-0	1243	1243	MS, RI, S
Furfuryl alcohol	98-00-0	1680	1661	MS, RI, O
Dimethyl trisulfide	3658-80-8	1366	1362	MS, RI
Trimethylpyrazine	14667-55-1	1408	1406	MS, RI, O
2,6-Diethylpyrazine	13067-27-1	1440	1444	MS, RI, O

RI exp. = experimentally determined retention index; RI lit. = retention index reported in the literature. MS = mass spectral matching; RI = retention index comparison; O = GC–O detection; S = identity confirmed using an authentic reference standard.
